# Supplementary material for: Provider perspectives on screening and treatment for opioid use disorder and mental health in HIV care: A qualitative study
Source: PLoS One. 2024 Jun 24;19(6):e0305174. doi: 10.1371/journal.pone.0305174 (PMC11195992; doi:10.1371/journal.pone.0305174)
Supplement: Attachment — Submitted filename: PONE-D-24-07882_Review corrections.pdf [file pone.0305174.s001.pdf]

PLOS ONE

Exploring strategies for integrating screening and treatment for opioid use disorder and mental health in HIV Care: A qualitative study with providers

--Manuscript Draft--

**Manuscript Number:**	PONE-D-24-07882
**Article Type:**	Research Article
**Full Title:**	Exploring strategies for integrating screening and treatment for opioid use disorder and mental health in HIV Care: A qualitative study with providers
**Short Title:**	Strategies for integrating screening and treatment for opioid use disorder and mental health in HIV Care
**Corresponding Author:**	Brandon A. Knettel<br>Duke University School of Nursing<br>Durham, NC UNITED STATES
**Keywords:**	Opioid epidemic; Human Immunodeficiency Virus (HIV); medication for opioid use disorder (MOUD); mental health; Harm Reduction; medication assisted treatment (MAT)
**Abstract:**	Background: HIV, opioid use disorder (OUD), and mental health challenges share multiple syndemic risk factors. Each can be effectively treated with routine outpatient appointments, medication management, and psychosocial support, leading implementers to consider integrated screening and treatment for OUD and mental health in HIV care. Provider perspectives are crucial to understanding barriers and strategies for integration.<br>Methods: We conducted in-depth qualitative interviews with 21 HIV treatment providers and social services providers to understand the current landscape, goals, and priorities for integrated OUD, mental health, and HIV care. Data were analyzed using applied thematic analysis in the NVivo 12 software program and evaluated for inter-coder agreement.<br>Results: Participants viewed substance use and mental health challenges as prominent barriers to engagement in HIV care. However, few organizations have integrated structured screening for substance use and mental health into their standard of care. Even fewer screen for opioid use. Although medication assisted treatment (MAT) is effective for mitigating OUD, providers struggle to connect patients with MAT due to limited referral options, social barriers such as housing and food insecurity, overburdened staff, stigma, and lack of provider training. Providers believed there would be clear benefit to integrating OUD and mental health treatment in HIV care but lacked resources for implementation.<br>Conclusions: Integration of screening and treatment for substance use and mental health in HIV care could mitigate many current barriers to treatment for all three conditions. Efforts are needed to train HIV providers to provide MAT, expand resources, and implement best practices.
**Order of Authors:**	Brandon A. Knettel
	Hillary Chen
	Elena Wilson
	David Agor
	Mehri S McKellar
	Susan Reif
**Additional Information:**	
**Question**	**Response**
**Financial Disclosure**<br><br>Enter a financial disclosure statement that	Yes

Powered by Editorial Manager® and ProduXion Manager® from Aries Systems Corporation

describes the sources of funding for the work included in this submission. Review the submission guidelines for detailed requirements. View published research articles from *PLOS ONE* for specific examples.<br><br>This statement is required for submission and **will appear in the published article** if the submission is accepted. Please make sure it is accurate.<br><br>**Funded studies**<br>Enter a statement with the following details:<br>• Initials of the authors who received each award<br>• Grant numbers awarded to each author<br>• The full name of each funder<br>• URL of each funder website<br>• Did the sponsors or funders play any role in the study design, data collection and analysis, decision to publish, or preparation of the manuscript?<br><br>Did you receive funding for this work?	
Please add funding details.<br>as follow-up to "**Financial Disclosure**<br><br>Enter a financial disclosure statement that describes the sources of funding for the work included in this submission. Review the submission guidelines for detailed requirements. View published research articles from *PLOS ONE* for specific examples.<br><br>This statement is required for submission and **will appear in the published article** if the submission is accepted. Please make sure it is accurate.	This work was supported by a grant from the Duke Center for AIDS Research, an NIH funded program (5P30 AI064518). We acknowledge additional support from the Duke School of Medicine Opioid Collaboratory, The Duke Global Health Institute's Center for Health Policy & Inequalities Research, and the Duke University School of Nursing Center for Nursing Research. The funders of the study had no role in study design, data collection, data analysis, data interpretation, or writing of the report.

**Funded studies**<br>Enter a statement with the following details:<br>• Initials of the authors who received each award<br>• Grant numbers awarded to each author<br>• The full name of each funder<br>• URL of each funder website<br>• Did the sponsors or funders play any role in the study design, data collection and analysis, decision to publish, or preparation of the manuscript?<br><br>Did you receive funding for this work?"	
Please select the country of your main research funder (please select carefully as in some cases this is used in fee calculation).<br>as follow-up to "**Financial Disclosure**<br><br>Enter a financial disclosure statement that describes the sources of funding for the work included in this submission. Review the submission guidelines for detailed requirements. View published research articles from *PLOS ONE* for specific examples.<br><br>This statement is required for submission and **will appear in the published article** if the submission is accepted. Please make sure it is accurate.<br><br>**Funded studies**<br>Enter a statement with the following details:<br>• Initials of the authors who received each award<br>• Grant numbers awarded to each author<br>• The full name of each funder<br>• URL of each funder website<br>• Did the sponsors or funders play any role in the study design, data collection and analysis, decision to publish, or preparation of the manuscript?<br><br>Did you receive funding for this work?"	UNITED STATES - US
**Competing Interests**	The authors have declared that no competing interests exist.

Use the instructions below to enter a competing interest statement for this submission. On behalf of all authors, disclose any competing interests that could be perceived to bias this work—acknowledging all financial support and any other relevant financial or non-financial competing interests.<br><br>This statement is required for submission and **will appear in the published article** if the submission is accepted. Please make sure it is accurate and that any funding sources listed in your Funding Information later in the submission form are also declared in your Financial Disclosure statement.<br><br>View published research articles from *PLOS ONE* for specific examples.<br><br>**NO authors have competing interests**<br><br>Enter: *The authors have declared that no competing interests exist.*<br><br>**Authors with competing interests**<br><br>Enter competing interest details beginning with this statement:<br><br>*I have read the journal's policy and the authors of this manuscript have the following competing interests: [insert competing interests here]*<br><br>* typeset	
**Ethics Statement**<br><br>Enter an ethics statement for this submission. This statement is required if the study involved:<br><br>• Human participants<br>• Human specimens or tissue<br>• Vertebrate animals or cephalopods<br>• Vertebrate embryos or tissues	The study was determined exempt by the Institutional Review Board of the Duke University Health System under Protocol #Pro00109790. Written informed consent was obtained from all participants at the time of enrollment.

Powered by Editorial Manager® and ProduXion Manager® from Aries Systems Corporation

• Field research<br><br>Write "N/A" if the submission does not require an ethics statement.<br><br>General guidance is provided below. Consult the submission guidelines for detailed instructions. **Make sure that all information entered here is included in the Methods section of the manuscript.**	

Powered by Editorial Manager® and ProduXion Manager® from Aries Systems Corporation

**Format for specific study types**<br><br>**Human Subject Research (involving human participants and/or tissue)**<br>• Give the name of the institutional review board or ethics committee that approved the study<br>• Include the approval number and/or a statement indicating approval of this research<br>• Indicate the form of consent obtained (written/oral) or the reason that consent was not obtained (e.g. the data were analyzed anonymously)<br><br>**Animal Research (involving vertebrate animals, embryos or tissues)**<br>• Provide the name of the Institutional Animal Care and Use Committee (IACUC) or other relevant ethics board that reviewed the study protocol, and indicate whether they approved this research or granted a formal waiver of ethical approval<br>• Include an approval number if one was obtained<br>• If the study involved *non-human primates*, add *additional details* about animal welfare and steps taken to ameliorate suffering<br>• If anesthesia, euthanasia, or any kind of animal sacrifice is part of the study, include briefly which substances and/or methods were applied<br><br>**Field Research**<br><br>Include the following details if this study involves the collection of plant, animal, or other materials from a natural setting:<br>• Field permit number<br>• Name of the institution or relevant body that granted permission	
**Data Availability**<br><br>Authors are required to make all data underlying the findings described fully available, without restriction, and from the time of publication. PLOS allows rare exceptions to address legal and ethical concerns. See the PLOS Data Policy and FAQ for detailed information.	No - some restrictions will apply

A Data Availability Statement describing where the data can be found is required at submission. Your answers to this question constitute the Data Availability Statement and **will be published in the article**, if accepted.<br><br>**Important:** Stating 'data available on request from the author' is not sufficient. If your data are only available upon request, select 'No' for the first question and explain your exceptional situation in the text box.<br><br>Do the authors confirm that all data underlying the findings described in their manuscript are fully available without restriction?	
**Describe where the data may be found in full sentences. If you are copying our sample text, replace any instances of XXX with the appropriate details.**<br><br>• If the data are **held or will be held in a public repository**, include URLs, accession numbers or DOIs. If this information will only be available after acceptance, indicate this by ticking the box below. For example: *All XXX files are available from the XXX database (accession number(s) XXX, XXX.)*.<br>• If the data are all contained **within the manuscript and/or Supporting Information files**, enter the following: *All relevant data are within the manuscript and its Supporting Information files.*<br>• If neither of these applies but you are able to provide **details of access elsewhere**, with or without limitations, please do so. For example:<br><br>*Data cannot be shared publicly because of [XXX]. Data are available from the XXX Institutional Data Access / Ethics Committee (contact via XXX) for researchers who meet the criteria for access to confidential data.*<br><br>*The data underlying the results presented in the study are available from (include the name of the third party*	A de-identified dataset may be made available upon request from the corresponding author after obtaining an appropriate data transfer agreement. Requests may be directed to the corresponding author. The data may also be made available upon request to Sharron L. Docherty, Vice Dean of the Center for Nursing Research at the Duke University School of Nursing, at doche002@mc.duke.edu or 919-668-3836.

and contact information or URL).<br>• This text is appropriate if the data are owned by a third party and authors do not have permission to share the data.<br><br>* typeset	
Additional data availability information:	

Manuscript
Click here to access/download;Manuscript;Opioid HIV Manuscript_26Feb2024.docx

Exploring strategies for integrating screening and treatment for opioid use disorder and mental health in HIV Care: A qualitative study with providers

Brandon A. Knettel [a,b]
Hillary Chen [c]
Elena Wilson [d]
David Agor [e]
Mehri S. McKellar [f]
Susan Reif [b,d]

[a] Duke University School of Nursing, Durham, NC
[b] Duke Global Health Institute, Duke University, Durham, NC
[c] Duke Department of Population Health Sciences, Durham, NC
[d] Center for Health Policy & Inequalities Research, Duke University, Durham, NC
[e] University of Pennsylvania School of Nursing, Philadelphia, PA
[f] Duke University School of Medicine, Durham, NC

Corresponding author: Correspondence concerning this article should be addressed to Brandon Knettel, Duke University School of Nursing, 307 Trent Dr., Durham, NC 27710. Phone: 919-660-1218. Email: brandon.knettel@duke.edu.

Conflict of Interest Statement: The authors declare no conflicts of interest in relation to this work.

Data Availability: The de-identified data for this manuscript may be made available upon reasonable request to the authors with the completion of an appropriate data transfer agreement.

Abstract

Background: HIV, opioid use disorder (OUD), and mental health challenges share multiple syndemic risk factors. Each can be effectively treated with routine outpatient appointments, medication management, and psychosocial support, leading implementers to consider integrated screening and treatment for OUD and mental health in HIV care. Provider perspectives are crucial to understanding barriers and strategies for integration.

Methods: We conducted in-depth qualitative interviews with 21 HIV treatment providers and social services providers to understand the current landscape, goals, and priorities for integrated OUD, mental health, and HIV care. Data were analyzed using applied thematic analysis in the NVivo 12 software program and evaluated for inter-coder agreement.

Results: Participants viewed substance use and mental health challenges as prominent barriers to engagement in HIV care. However, few organizations have integrated structured screening for substance use and mental health into their standard of care. Even fewer screen for opioid use. Although medication assisted treatment (MAT) is effective for mitigating OUD, providers struggle to connect patients with MAT due to limited referral options, social barriers such as housing and food insecurity, overburdened staff, stigma, and lack of provider training. Providers believed there would be clear benefit to integrating OUD and mental health treatment in HIV care but lacked resources for implementation.

Conclusions: Integration of screening and treatment for substance use and mental health in HIV care could mitigate many current barriers to treatment for all three conditions. Efforts are needed to train HIV providers to provide MAT, expand resources, and implement best practices.

Keywords: Opioid epidemic, HIV, medication for opioid use disorder (MOUD), mental health, harm reduction, medication assisted treatment (MAT)

INTRODUCTION

In 2021, opioid overdose contributed to 80,816 deaths in the United States, making it the leading cause of injury death in the nation (Centers for Disease Control and Prevention, 2022, 2018). In addition, opioid misuse can impact well-being, increasing the risk for homelessness, unemployment, mental health challenges, and sexually transmitted infections, including HIV (Baldini et al., 2012; Hollingsworth et al., 2017; López-Martínez et al., 2019; SAMHSA, 2018; Yamamoto et al., 2019). Relationships between HIV, opioid use disorder (OUD), and mental health challenges are complex and syndemic. People living with HIV (PLWH) are more likely to experience chronic pain compared to uninfected people, even when their HIV is well managed, likely due to chronic inflammation and social challenges that are more common among PLWH; one study found that nearly half (48.7%) of PLWH struggle with daily pain (American Psychiatric Association, 2012).

Because of challenges with chronic pain, PLWH are more likely to be treated with opioids, creating increased risk for addiction (Cunningham, 2018; Perlman and Jordan, 2018). Simultaneously, PLWH who use opioids are more likely to experience challenges across the HIV care continuum, including delayed entry into care, suboptimal linkage to treatment, and lower adherence to antiretroviral therapy (Cernasev et al., 2020). HIV and OUD also share multiple social determinants – including low health literacy, poverty, and exposure to violence – that can contribute to poor treatment outcomes such as increased disability, morbidity, and mortality (Altekruse et al., 2020; Beltran et al., 2022).

States in the U.S. South have been disproportionately impacted by both HIV and opioid use disorder (Schranz et al., 2021). Mecklenburg County, North Carolina (NC) is designated as a priority jurisdiction in the national plan for Ending the HIV Epidemic (EHE) (HIV.gov, 2021), with HIV prevalence nearly three times higher than state and national rates (AIDSVu, 2018). The county also faces a considerable burden in the opioid epidemic, with an estimated 165 opioid overdose deaths in 2020, which is by far the greatest of any substance measured (North

Carolina Department of Health and Human Services, 2023). Yearly opioid overdose deaths in North Carolina more than quadrupled from 2012 to the end of 2021, including a dramatic spike during the COVID-19 pandemic (North Carolina Department of Health and Human Services, 2023). Mecklenburg is a large county that includes the greater Charlotte metropolitan area, surrounding suburban areas, and several rural communities, each of which faces unique challenges in improving access to screening and treatment for both HIV and OUD.

The most effective intervention for OUD is medication-assisted treatment (MAT) combining medications for opioid use disorder such as buprenorphine, naltrexone, and methadone, along with psychosocial therapy (Amato et al., 2011; Ma et al., 2019; Moore et al., 2019; Thomas et al., 2014). HIV and OUD are both chronic conditions that are treatable with daily medication, with outcomes enhanced by strong psychosocial support (Sanders et al., 2023; Wen et al., 2023). Further, both HIV and OUD have frequently been linked to mental health challenges; for example, experiencing prior trauma is a risk factor for both HIV and OUD, while other conditions including depression and anxiety may be present before HIV infection or the onset of opioid use, or may develop after a diagnosis (Bhatraju et al., 2023; Caniglia et al., 2021; Glynn et al., 2019a).

The common comorbidity of HIV, OUD, and mental health challenges have led implementers to consider integrated treatment for these conditions under one roof, and some efforts have been made to improve access to mental health care in the context of HIV care (Caniglia et al., 2021; Oldfield et al., 2019). A systematic review identified six studies examining the integration of treatment for OUD in HIV care settings, five of which focused on MAT and one of which offered counseling only (Oldfield et al., 2019). These studies demonstrated several positive clinical benefits for both HIV and OUD, included improved initiation of antiretroviral therapy (ART), decreased opioid use, decreased needle sharing, and improved health-related quality of life (Oldfield et al., 2019). Another recent review identified benefits for HIV viral suppression (Hill et al., 2023). However, the integration of OUD treatment in HIV care did

require considerable investment, including commitment from leadership to support multidisciplinary care teams, up-to-date provider training, and sufficient pharmacy stock for substance use treatment (Hill et al., 2023). Additional barriers included added costs for labor, facilities, and urine toxicology testing, as well as a higher burden of treatment for existing HIV specialists (Oldfield et al., 2019). Although studies have emerged demonstrating the benefits of integrated OUD treatment in HIV care, few have addressed the common comorbidity with mental health challenges.

The objectives of this study were to further explore the potential benefits, barriers, and strategies for the implementation of opioid screening and treatment, along with mental health treatment, in HIV care. To achieve these objectives, we interviewed HIV treatment providers and social service providers who are currently offering services related to HIV and OUD in Mecklenburg Country, North Carolina.

METHODS

The study team conducted in-depth qualitative interviews with a purposive sample of medical and social service providers offering treatment for people living with HIV and OUD in Mecklenburg County, North Carolina. The team first compiled a list of known local providers engaged in HIV-related care and/or OUD treatment, and contacted each to invite them to participate in a focus group or individual qualitative interview. HIV-related care included HIV testing, antiretroviral medication management for PLWH, and pre-exposure prophylaxis (PrEP) for people with elevated risk of HIV infection. Participants were asked to name other providers in the county and these providers were then contacted and invited to participate. We reached out to 15 providers, all of whom either completed an interview or referred us to other professionals within their organization who completed an interview.

A member of the research study team trained in qualitative interviewing conducted 30-60 minute virtual in-depth individual interviews (IDIs) with 12 providers using a semi-structured

interview guide on videoconferencing software. Nine additional participants at one large site participated in a virtual group interview. Interviews took place between May 3, 2022 and October 24, 2022. Prior to each interview, participants completed a brief verbal questionnaire to collect demographic and background information including their professional experience, current role, and characteristics of their organization. All participants signed an online consent form prior to participating. No compensation was provided for participation.

The interview guide provided language for initial questions and prompts for additional probes, as well as flexibility for the interviewer to probe additional lines of inquiry based on their judgment. All interviews were audio recorded, transcribed and de-identified, and uploaded to a secure online database for analysis. The study was determined exempt by the Institutional Review Board of the Duke University Health System.

The Qualitative Interview Guide

The development of the interview guide was informed by the Implementation Research Logic Model (IRLM) (Powell et al., 2019; Smith et al., 2020), which considers contextual determinants, including compatibility with existing care models and stakeholder acceptability, strategies to address identified barriers, and mechanisms for outcome improvement. Data from the study are intended to inform future implementation strategies to improve OUD and mental health care in the context of HIV treatment, which may include OUD/stigma education and harm reduction training for HIV clinic personnel, introducing OUD and mental health screening in HIV care, integrating mental health treatment into care, and training HIV clinic providers to offer MAT. Additional detail on the interview guide, with associated IRLM elements, can be found in Table 1.

Table 1. Summary of Semi-Structured Interview Guide

Domain	Area of Inquiry	IRLM Core Element(s)	Questions and Prompts
Patient Characteristics	Patient population	Implementation Determinants	• Population served<br>• Mental health among patients

			• Substance use among patients<br>• Opioid use among patients<br>• Patient interest in MAT<br>• Challenges to treatment access
Current Clinic Services Related to HIV, Substance Use, and Mental Health	HIV testing, treatment and services currently offered	Implementation Determinants	• Treatment and services currently offered at the clinic<br>• Treatment and services offered elsewhere in the community<br>• Referral process and challenges
	Screening for substance use	Implementation Strategies and Mechanisms	• Whether screening tool is used<br>• Which patients screened<br>• Who administers screening<br>• Screening follow up<br>• Patient comfort being screened
	Substance use treatment and services currently offered	Implementation Determinants	• Treatment and services currently offered at the clinic (if any)<br>• Treatment and services offered elsewhere in the community<br>• Referral process and challenges
	Mental health treatment and services currently offered	Implementation Determinants	• Screening, treatment, and services currently offered at the clinic (if any)<br>• Treatment and services offered elsewhere in the community<br>• Referral process and challenges
	Strategies and resources needed to improve or expand services	Implementation Strategies and Mechanisms	• Substance use and mental health screening<br>• To improve or expand existing services<br>• To improve referral processes for external services<br>• To offer new services

			• To support clinic with resources and provider training to offer MAT
Provider experiences related to MAT in the context of HIV-related care		Implementation Mechanisms and Outcomes	• Provider training<br>• Motivation/interest in offering MAT<br>• Challenges to providing MAT<br>• Benefits of providing MAT<br>• Clinic support for offering MAT

Note. MAT: medication-assisted treatment for opioid use disorder

Qualitative Analysis

Prior to commencing recruitment, the research team discussed personal reflexivity, including personal attributes and prior knowledge, experience, and assumptions related to HIV, OUD, and mental health. The research team included content experts in each of these areas, and we conducted a one-hour team training on cultural humility, stigma, and harm reduction principles in research. Interviews were analyzed using an applied thematic approach (Guest et al., 2012) and NVivo 12, a qualitative data software program, to code the data. Thematic data analysis included a combination of deductive and inductive coding, with a priori themes based on the qualitative interview guide utilized for the development of broader categories in the codebook with the emergence of subcodes developed from rereading the collected data transcripts. Two interviews were initially coded by multiple team members, who then came together to discuss and refine the codebook, add emergent theme categories, and resolve any discrepancies of interpretation of the data to ensure validity. The remainder of interviews were then coded using line by line analysis.

The study sample size was determined by the number of eligible providers in Mecklenburg County and considerations of data saturation, the number of new themes emerging in new interviews during preliminary analysis (Guest et al., 2006).

We randomly selected three individual interviews and the group interview to be re-coded by a second reviewer and evaluated for inter-coder agreement using a pre-established threshold of 80% agreement (Campbell et al., 2013). Inter-coder agreement on these transcripts was 87.7% (range 85.7-88.9), which exceeded the desired threshold.

RESULTS

Interview Participants

Interviews were conducted with 21 health care and social service providers currently offering services related to HIV and/or substance use at 11 unique facilities in Mecklenburg Country, NC. We conducted one group interview with 9 participants and 12 individual interviews with 15 social services providers and 6 medical providers, including outreach workers, social workers, case managers, clinical program coordinators, peer specialists, outreach workers, clinic directors, medical doctors, and a physician assistant. The majority of participants (67%) were ages 30-49 years, with more representation (62%) from people identifying as female than male and two-thirds (67%) identifying as Black/African American. Interviewees had been in their current role for an average of 5 years, with a range of 1 to 15 years (Table 2).

Table 2. Participant Demographics (N=21)

Demographic Characteristics	n (%)
**Gender**	
Female	13 (62%)
Male	8 (38%)
**Age**	
18-29	1 (5%)
30-49	14 (67%)
50-64	6 (29%)
**Race**	
Black or African American	14 (67%)
Non-Hispanic white	6 (29%)
Both white and Black or African American	1 (5%)

Organizational Characteristics

Participants provided information on characteristics of the clinic or organization where they work. These included five community nonprofit organizations that provide HIV treatment and prevention services, two Federally Qualified Health Centers (FQHCs), two clinics that provide both primary care and infectious diseases care, one hospital infectious diseases clinic, and the county Health Department. HIV-related care typically included HIV testing, antiretroviral medication management for PLWH, pre-exposure prophylaxis (PrEP) for people with elevated risk of HIV, and management of common comorbid health conditions such as hepatitis. At several clinics, HIV providers also offered primary medical care for conditions unrelated to HIV. All of the clinics work with uninsured patients and almost all accept Medicaid/Medicare.

Only one clinic offered in-house MAT for patients with OUD, in the form of sublingual buprenorphine/naloxone, which was also a clinic that offered both primary care and infectious diseases care. A provider at this clinic noted “increasing amounts of substance use in general and increasing demand for our MAT program,” with 30% growth in their MAT patient population in the prior year. As demand has increased, they have hired more providers capable of providing MAT to help meet this need. All of the clinics that did not offer in-house services did offer linkages or referral to outside services such as housing assistance, mental health, substance use treatment, food support, and harm reduction services. No participants reported having peer support workers at their clinic. Roughly half had social workers and/or case managers.

With regard to mental health treatment, three clinics had an in-house provider whereas about half of participants described referring patients with mental health needs to outside clinics or community organizations that can provide needed care. Most participants felt there would be a clear benefit to adding more integrated services within their clinic, including support for substance use mental health treatment, and MAT. The primary barriers to adding these services were lack of financial support and human resources to implement new programs. For example,

several participants mentioned actively seeking to hire providers to offer mental health care in-house, but experiencing challenges in finding candidates for these positions. As a result, most clinics currently prioritize providing HIV-related care and treatment specifically.

Substance Use Among Patients Engaged in HIV-Related Care

Nearly all of the study participants described substance use and mental health challenges as common among patients seeking HIV-related care. Most interviewees highlighted alcohol, marijuana, and sometimes methamphetamine and crack or cocaine as substances they see most frequently among the populations they serve, whereas opioids were emphasized less often. When asked specifically about opioid use among their patients, many participants focused their responses on injection opioid use.

> *“In regards to substance abuse, crack and cocaine have always been a thing and heroin has always been a thing. Marijuana has always been a thing. But as the years have gone by, you saw people mixing different drugs together and they're more addictive in less time. So a lot of times, when you're talking to clients, things that they used to be able to pull away from, they can no longer pull away from.”*

When asked about other routes of administration including misuse of prescription opioid medications, many participants acknowledged that this is likely a much more common, but hidden, problem.

Despite the perception that drug use is very common among their patients, more than half of participants stated that they do not assess for substance use during HIV-related appointments or that assessment is not done consistently. Among those who do assess for substance use, most stated they do not use formal assessments, but ask the questions informally or that they only ask about substance use if it seems relevant to the patient’s HIV care. For example, one participant stated, it is a “clinician-to-clinician decision about how they

like to screen and talk about [treatment] options." More than half of participants identified improving or standardizing their screening procedures as an area for improvement of services.

Among patients who use drugs, participants expressed that there is commonly a lack of awareness and acceptance among patients of the need for treatment to manage their use. When asked whether specific subsets of their patient population are more likely to be experiencing challenges related to substance use, several participants indicated that sexual and gender minority (SGM [LGBTQIA+]) patients (especially men who have sex with men [MSM] and transgender individuals) and unhoused patients are groups that seem to be disproportionately affected by substance use.

Other Challenges Influencing Engagement with HIV-Related Care

Nearly all of the participants described mental health challenges as common among patients in HIV-related care and reported that mental health challenges often co-occur with substance use. Common mental health challenges included depression, posttraumatic stress, and anxiety. Participants also described seeing patients with cognitive impairment and psychosis, both of which can occur due to chronic drug use.

> *"[Our patients] are very chronic, long-term substance users oftentimes, and these are usually individuals that also may have cognitive, as well as mental health diagnoses, which makes it a lot more challenging for them to manage all of that at the same time."*

Participants described mental health challenges as having a significant negative impact on HIV care engagement, including disruptions to long-term retention in care, consistency in attending appointments, and adherence to antiretroviral medication and other treatments. This created a cyclical pattern of deteriorating physical health and mental health that, especially when coupled with substance use, could lead to likely increases in morbidity and mortality.

Participants identified several other common barriers to HIV patients' engagement with treatment and overall health, often related to finances and social challenges. One participant

described it in this way; “When you take mental health and substance use, you’ve got the main barriers for our patients, followed by housing insecurity, food insecurity, transportation, and then criminal justice involvement.”

Participants noted there is a lack of clinic and community resources available to support individuals with social challenges such as housing insecurity. Some clinics provide support to coordinate and link patients to housing, prevent patients from being evicted from their homes, assist with food, or to cover utility costs. Several interviewees explained that without stable housing, patients with HIV, substance use disorders, and other mental health challenges often struggle to stay consistently engaged in their HIV-related care and to seek substance use or mental health treatment.

> *“The resources [for housing] just aren’t there in a lot of cases… It’s kind of like the hierarchy of needs. Without housing, it’s difficult for them to seek mental health services or substance use services because their main concern at that point in time is housing. Even their health goes on the back burner because they just want a place to stay.”*

Many participants also highlighted transportation as a significant barrier to accessing services. Some noted that their clinics can offer transportation vouchers, cover costs using outside funding, or offer services via telemedicine to ameliorate this challenge. Nevertheless, transportation challenges continue to be a common reason provided by patients for missing appointments or falling out of treatment.

Several participants discussed financial barriers to care, noting that some clinics in the area do not accept patients without insurance, do not provide Medicaid/Medicare coverage, or require payment beyond patients’ capabilities even with sliding scales. These financial challenges extend to medication prescription coverage, as well as substance use and mental health care. Interview participants explained that though there are a few social services programs in the area that subsidize copays for mental health care or substance use or provide

services free of charge, availability is extremely limited and has inclusion criteria for qualification.

Finally, more than half of the participants described a negative impact of stigma related to mental health and substance use that can prevent patients from discussing substance use or mental health challenges with providers or other support systems, or from asking about services that may be available to them. A few interviewees described stigmatizing comments they have heard or behaviors they have seen staff engage in and noted that this can negatively impact patients' experiences in care. Multiple participants identified cultural humility and stigma reduction as areas in which their organizations could benefit from training.

Areas to Improve Substance Use and Mental Health Treatment in HIV-Related Care

More than half of participants discussed challenges related to referring patients for mental health and/or substance use services. Some of these challenges include long waitlists, difficulty following up with patients to ensure they were able to connect to care, lack of available services to refer to (especially for patients with financial barriers), and the burden of building trust with another provider that patients may engage with when being referred out for care. Given these challenges with referrals, some HIV providers attempted to provide counseling and prescribe psychotropic medications, but they often encountered cases they did not have the expertise or capacity to manage in-house.

> *"There is a significant number of people with untreated mental health issues, and our providers are not comfortable treating anything more than like minor depression. They're okay putting them on like a low dose SSRI. They're not doing any kind of full psychiatric evaluation and they're definitely not prescribing anti-psychotic drugs."*

Participants acknowledged that the lack of referral options was a complex problem that required large scale, systems-level interventions, such as increased government support to increase the

availability of substance use and mental health services and improve access to existing treatment options.

With limited external referral options, several interviewees noted how beneficial it is when a clinic can provide HIV-related care, primary care, mental health, and substance use treatment all under one roof.

> *"It would be great if substance use [treatment] was in-house for us, because when we have a client that is interested in treatment that tends to be where we lose them, because they don't want to have to go to this agency and that agency and this place and that place. If they could just come to a central location with us, I think that we would have a bit more success in getting clients to engage."*

Nearly all of the participants discussed the need for more provider training to enhance knowledge on how to treat addiction, raise awareness of services available in the community, increase cultural humility, and reduce stigma when working with patients experiencing substance use and/or mental health challenges.

> *"More training. That is definitely needed. Sensitivity training, especially towards substance use. I hear a little undercurrent every now and then where people make comments about drug use, as if it is some deliberate diagnosis that they have a choice in. Everybody needs it."*

Participants felt that providers would be open to more education. However, many interviewees also expressed concern about current staff workloads and the need for more staff to take on new initiatives focused on improving linkage to substance use and mental health treatment.

Several participants explained that having peer navigators to support patients would be beneficial for facilitating trust, helping patients to access needed services, and successfully referring patients for mental health and substance use care.

"I think that [patients] would be definitely more comfortable talking to people in outreach or somebody who's a peer that can just talk about their experiences…I think that people are more likely to open up if it's somebody who they know is in the community as well."

DISCUSSION

In these interviews with healthcare and social services professionals providing HIV-related care, there was strong consensus about the challenges of comorbid substance use and mental health challenges, the need for improved services in these areas, and the benefit of integrated HIV, substance use, and mental health treatment under one roof. There is an emerging evidence base demonstrating the value of integrated substance use treatment in HIV care (Hill et al., 2023; Oldfield et al., 2019); however, efforts at implementing these findings in routine clinical care remain nascent and little attention has been paid to syndemic patterns of HIV, substance use, and other mental health challenges.

The U.S. government's plan for Ending the HIV Epidemic (EHE), introduced in 2019, includes a goal of a 90% reduction of new HIV infections in the U.S. by 2030 (HIV.gov, 2021). To achieve this goal, the plan includes an emphasis on priority populations and jurisdictions with high HIV prevalence, increases in funding for HIV prevention and treatment, and addressing key barriers to progress in ending the epidemic, "including trends in injection and other drug use; HIV-related stigma; homonegativity and transnegativity; lack of access to HIV prevention, testing, and treatment; and a lack of awareness that HIV remains a significant public health threat" (HIV.gov, 2021). The site of this research, Mecklenburg County, North Carolina, was identified as one of 48 counties with the highest number of new HIV diagnoses to be prioritized in the EHE plan (HIV.gov, 2021).

In the current research, substance use was described as extremely common among patients seeking HIV-related services, with a rapidly increasing burden of OUD in this

population. However, few of the participants' clinics conducted standardized evidence-based screening for substance use with valid psychometric scales, and only one clinic provided in-house substance use treatment and MAT. These findings add to a large and growing body of literature highlighting the challenge of OUD in HIV care and failures of the health system to adequately respond by improving access to OUD treatment (Cernasev et al., 2020; Critchley et al., 2020; Waters et al., 2021). MAT is an effective treatment for OUD, involving daily medication to reduce opioid cravings, supplemented by behavioral support, adherence counseling, and education (Chen et al., 2023; National Institute on Drug Abuse, 2021). Prior to 2023, providers required specific training and an associated waiver to prescribe medications for OUD, such as buprenorphine; however, these requirements have now been waived, removing a crucial barrier to providers in all settings offering these safe and effective treatments (SAMHSA, 2023). It will be crucial in the coming months to capitalize on the improved regulatory environment by seeking to bring evidence-based models for OUD treatment to scale within HIV treatment settings.

Mental health treatment is another crucial aspect of care that can improve quality of life, reduce risk of acquiring HIV or initiating substance use, and improve outcomes of PLWH and people who use opioids (Ikekwere et al., 2023; Wyse et al., 2022). In the current study, participants reported barriers to mental health care that hinder treatment for HIV and substance use, with few clinics offering formal mental health screening and limited in-house options for treatment. These challenges were further exacerbated by poor external referral options for mental health care, including a lack of providers, long wait times, and high costs of treatment. Further, participants expressed concerns about HIV stigma that might occur in the health system and the desire to know and trust providers before sending referrals to them. For PLWH who are also struggling with a substance use disorder and/or another mental health disorder, each condition brings the potential for stigma that can impact social support, disclosure, care engagement, and health outcomes (Armoon et al., 2022; Levi-Minzi and Surratt, 2014).

Participants identified several common social concerns that often co-occurred with HIV, substance use, and mental health challenges, including housing insecurity, food insecurity, and criminal justice involvement. Whether these challenges existed prior to receiving an HIV diagnosis or arise afterward, they must be addressed to increase the likelihood of long-term treatment adherence and positive health outcomes (Beyrer et al., 2021; Carrico, 2019; Glynn et al., 2019b). Both HIV care and OUD treatment rely strongly on attention to behavioral health, daily medication adherence, health education, and addressing social determinants to maximize the potential for treatment success (Carrico, 2019; Glynn et al., 2019b). Therefore, it is logical, resource efficient, and imperative to combine treatment approaches for HIV and OUD under one roof (Fanucchi et al., 2019; Hill et al., 2023; Oldfield et al., 2019).

Participants in our study identified integrated treatment for HIV, OUD, and mental health as valuable and desirable, but also difficult to implement due to current providers and staff feeling overburdened and lacking resources to develop new programs. At the national level, some important steps have been made to open the door for improved treatment integration. These include the elimination of the waiver requirement for prescribing medication for OUD (SAMHSA, 2023), new government funding and drug company settlements to address the opioid epidemic (U.S. Department of Health & Human Services, 2022), and increased support for HIV treatment through the EHE plan (HIV.gov, 2021). However, sustained efforts are needed to ensure this support reaches providers and patients through evidence-based interventions, and integrated treatments among high-risk groups should be at the top of this list. This will require investments at all levels of the healthcare workforce to increase treatment capacity, including the task-sharing, task-shifting, to peer navigators and community health workers (Rogers et al., 2020). Telehealth treatment may also be a promising direction to maximize resources (Chen et al., 2023; Oldfield et al., 2019; Rogers et al., 2020).

Findings from this study should be interpreted in light of the following limitations. We recruited participants from diverse organizations providing HIV-related services in Mecklenburg

County, North Carolina; however, perspectives may not be representative of the broader professional community in the state or elsewhere. As care providers were the subjects of these interviews, information related to HIV and OUD treatment participation for community members who are not engaged in care were not represented. Additionally, this analysis did not include the perspectives of people with lived experience related to HIV-related care, opioid use, or mental health or from primary opioid treatment providers, which will be crucial for the next phase of this research.

Conclusions

In this study, HIV care providers identified a high burden of comorbid substance use disorders and mental health challenges, but experienced multiple barriers in connecting patients to care for these challenges. These included syndemic social challenges faced by patients such as housing insecurity and difficulties with transportation, lack of appropriate referrals, and stigma within the health system. Few clinics offered integrated, in-house treatment options for OUD or mental health, often due to high burdens placed on current providers and lack of resources for new services. Future efforts must emphasize identifying strategies to overcome these barriers and implement evidence-based strategies for HIV, OUD, and mental health treatment integration.

References

AIDSVu, 2018. Local Data: Charlotte (Mecklenburg County) [WWW Document]. URL http://aidsvu.org/local-data/united-states/south/north-carolina/charlotte/ (accessed 5.7.21).

Altekruse, S.F., Cosgrove, C.M., Altekruse, W.C., Jenkins, R.A., Blanco, C., 2020. Socioeconomic risk factors for fatal opioid overdoses in the United States: Findings from the Mortality Disparities in American Communities Study (MDAC). PLoS One 15, e0227966. https://doi.org/10.1371/journal.pone.0227966

Amato, L., Minozzi, S., Davoli, M., Vecchi, S., 2011. Psychosocial and pharmacological treatments versus pharmacological treatments for opioid detoxification. Cochrane Database of Systematic Reviews. https://doi.org/10.1002/14651858.CD005031.pub4

American Psychiatric Association, 2012. Pain in HIV/AIDS. American Psychiatric Association, Washington, DC.

Armoon, B., Fleury, M.-J., Bayat, A.-H., Fakhri, Y., Higgs, P., Moghaddam, L.F., Gonabadi-Nezhad, L., 2022. HIV related stigma associated with social support, alcohol use disorders, depression, anxiety, and suicidal ideation among people living with HIV: a systematic review and meta-analysis. International Journal of Mental Health Systems 16, 17. https://doi.org/10.1186/s13033-022-00527-w

Baldini, A., Von Korff, M., Lin, E.H.B., 2012. A Review of Potential Adverse Effects of Long-Term Opioid Therapy: A Practitioner's Guide. Prim Care Companion CNS Disord 14. https://doi.org/10.4088/PCC.11m01326

Beltran, R.M., Holloway, I.W., Hong, C., Miyashita, A., Cordero, L., Wu, E., Burris, K., Frew, P.M., 2022. Social Determinants of Disease: HIV and COVID-19 Experiences. Curr HIV/AIDS Rep 19, 101–112. https://doi.org/10.1007/s11904-021-00595-6

Be[illegible], C., Adimora, A.A., Hodder, S.L., Hopkins, E., Millett, G., Mon, S.H.H., Sullivan, P.S., Walensky, R.P., Pozniak, A., Warren, M., Richman, B., Copeland, R., Mayer, K.H., 2021. Call to action: how can the US Ending the HIV Epidemic initiative succeed? The Lancet 397, 1151–1156. https://doi.org/10.1016/S0140-6736(21)00390-1

Bhatraju, E., Liebschutz, J.M., Lodi, S., Forman, L.S., Lira, M.C., Kim, T.W., Colasanti, J., del Rio, C., Samet, J.H., Tsui, J.I., 2023. Post-traumatic stress disorder and risky opioid use among persons living with HIV and chronic pain. AIDS Care 35, 1173–1180. https://doi.org/10.1080/09540121.2021.1876838

Campbell, J.L., Quincy, C., Osserman, J., Pedersen, O.K., 2013. Coding In-depth Semistructured Interviews: Problems of Unitization and Intercoder Reliability and Agreement. Sociological Methods & Research 42, 294–320. https://doi.org/10.1177/0049124113500475

Caniglia, E.C., Khan, M., Ban, K., Braithwaite, R.S., 2021. Integrating Screening and Treatment of Unhealthy Alcohol Use and Depression with Screening and Treatment of Anxiety, Pain, and Other Substance Use Among People with HIV and Other High-Risk Persons. AIDS Behav 25, 339–346. https://doi.org/10.1007/s10461-021-03245-5

Carrico, A.W., 2019. Getting to Zero: Targeting Psychiatric Comorbidities as Drivers of the HIV/AIDS Epidemic. Int.J. Behav. Med. 26, 1–2. https://doi.org/10.1007/s12529-019-09771-w

Centers for Disease Control and Prevention, 2022. U.S. Overdose Deaths In 2021 Increased Half as Much as in 2020 - But Are Still Up 15% [WWW Document]. URL https://www.cdc.gov/nchs/pressroom/nchs_press_releases/2022/202205.htm (accessed 7.17.23).

Centers for Disease Control and Prevention, 2018. Multiple Cause of Death, 1999-2018. Centers for Disease Control and Prevention National Center for Health Statistics.

Cernasev, A., Veve, M.P., Cory, T.J., Summers, N.A., Miller, M., Kodidela, S., Kumar, S., 2020. Opioid Use Disorders in People Living with HIV/AIDS: A Review of Implications for Patient Outcomes, Drug Interactions, and Neurocognitive Disorders. Pharmacy (Basel) 8. https://doi.org/10.3390/pharmacy8030168

Chen, H., Knettel, B.A., Harrison, L.M., Marais, A.D., Nelli, A., Campbell, H.A., Muhirwa, A., McKellar, M.S., Gulur, P., Guthrie, S., 2023. Leveraging Policy Changes Made in Response to COVID-19 to Advance Telehealth and Novel Treatment Approaches for People Who Use Opioids: Qualitative Perspectives From Two Rural North Carolina Counties. North Carolina Medical Journal 84. https://doi.org/10.18043/001c.83930

Critchley, L., Carrico, A., Gukasyan, N., Jacobs, P., Mandler, R.N., Rodriguez, A.E., del Rio, C., Metsch, L.R., Feaster, D.J., 2020. Problem opioid use and HIV primary care engagement among hospitalized people who use drugs and/or alcohol. Addiction Science & Clinical Practice 15, 19. https://doi.org/10.1186/s13722-020-00192-9

Cunningham, C.O., 2018. Opioids and HIV Infection: From Pain Management to Addiction Treatment. Top Antivir Med 25, 143–146.

Fanucchi, L., Springer, S.A., Korthuis, P.T., 2019. Medications for Treatment of Opioid Use Disorder among Persons Living with HIV. Curr HIV/AIDS Rep 16, 1–6. https://doi.org/10.1007/s11904-019-00436-7

Glynn, T.R., Llabre, M.M., Lee, J.S., Bedoya, C.A., Pinkston, M.M., O'Cleirigh, C., Safren, S.A., 2019a. Pathways to Health: an Examination of HIV-Related Stigma, Life Stressors, Depression, and Substance Use. Int.J. Behav. Med. 26, 286–296. https://doi.org/10.1007/s12529-019-09786-3

Glynn, T.R., Safren, S.A., Carrico, A.W., Mendez, N.A., Duthely, L.M., Dale, S.K., Jones, D.L., Feaster, D.J., Rodriguez, A.E., 2019b. High Levels of Syndemics and Their Association with Adherence, Viral Non-suppression, and Biobehavioral Transmission Risk in Miami, a U.S. City with an HIV/AIDS Epidemic. AIDS Behav 23, 2956–2965. https://doi.org/10.1007/s10461-019-02619-0

Guest, G., Bunce, A., Johnson, L., 2006. How Many Interviews Are Enough?: An Experiment with Data Saturation and Variability. Field Methods 18, 59–82. https://doi.org/10.1177/1525822X05279903

Guest, G., MacQueen, K.M., Namey, E.E., 2012. Applied Thematic Analysis. SAGE, Thousand Oaks, CA.

Hill, K., Kuo, I., Shenoi, S.V., Desruisseaux, M.S., Springer, S.A., 2023. Integrated Care Models: HIV and Substance Use. Curr HIV/AIDS Rep 20, 286–295. https://doi.org/10.1007/s11904-023-00667-9

HIV.gov, 2021. Ending the HIV Epidemic: A Plan for America [WWW Document]. URL https://www.hiv.gov/federal-response/ending-the-hiv-epidemic/overview (accessed 5.7.21).

Hollingsworth, A., Ruhm, C.J., Simon, K., 2017. Macroeconomic conditions and opioid use. National Bureau of Economic Research, Cambridge, MA.

Ikekwere, J., Eiger, R., Heward, B., Longcoy, J., Okobi, O.E., Oladunjoye, A.F., Onyeaka, H., Kompella, S., Jegede, O., 2023. Opioid use disorder amongst young people living with HIV: A 10-year trend. Psychiatry Research Communications 3, 100140. https://doi.org/10.1016/j.psycom.2023.100140

Levi-Minzi, M.A., Surratt, H.L., 2014. HIV Stigma Among Substance Abusing People Living with HIV/AIDS: Implications for HIV Treatment. AIDS Patient Care STDS 28, 442–451. https://doi.org/10.1089/apc.2014.0076

López-Martínez, A.E., Reyes-Pérez, Á., Serrano-Ibáñez, E.R., Esteve, R., Ramírez-Maestre, C., 2019. Chronic pain, posttraumatic stress disorder, and opioid intake: A systematic review. World J Clin Cases 7, 4254–4269. https://doi.org/10.12998/wjcc.v7.i24.4254

Ma, J., Bao, Y.-P., Wang, R.-J., Su, M.-F., Liu, M.-X., Li, J.-Q., Degenhardt, L., Farrell, M., Blow, F.C., Ilgen, M., Shi, J., Lu, L., 2019. Effects of medication-assisted treatment on mortality among opioids users: a systematic review and meta-analysis. Molecular Psychiatry 24, 1868–1883. https://doi.org/10.1038/s41380-018-0094-5

Moore, K.E., Roberts, W., Reid, H.H., Smith, K.M.Z., Oberleitner, L.M.S., McKee, S.A., 2019. Effectiveness of medication assisted treatment for opioid use in prison and jail settings: A meta-analysis and systematic review. J Subst Abuse Treat 99, 32–43. https://doi.org/10.1016/j.jsat.2018.12.003

National Institute on Drug Abuse, 2021. How effective are medications to treat opioid use disorder? [WWW Document]. National Institute on Drug Abuse. URL https://nida.nih.gov/publications/research-reports/medications-to-treat-opioid-addiction/efficacy-medications-opioid-use-disorder (accessed 9.12.23).

North Carolina Department of Health and Human Services, 2023. Statewide Overdose Deaths by Drug: NC Residents, 2012-2021. Raleigh, NC.

Oldfield, B.J., Muñoz, N., McGovern, M.P., Funaro, M., Villanueva, M., Tetrault, J.M., Edelman, E.J., 2019. Integration of care for HIV and opioid use disorder. AIDS 33, 873. https://doi.org/10.1097/QAD.0000000000002125

Perlman, D.C., Jordan, A.E., 2018. The syndemic of opioid misuse, overdose, HCV and HIV: Structural-level causes and interventions. Curr HIV/AIDS Rep 15, 96–112. https://doi.org/10.1007/s11904-018-0390-3

Powell, B.J., Fernandez, M.E., Williams, N.J., Aarons, G.A., Beidas, R.S., Lewis, C.C., McHugh, S.M., Weiner, B.J., 2019. Enhancing the Impact of Implementation Strategies in Healthcare: A Research Agenda. Front Public Health 7. https://doi.org/10.3389/fpubh.2019.00003

Rogers, B.G., Arnold, T., Schierberl Scherr, A., Strong, S.H., Holcomb, R., Daley Ndoye, C., Pinkston, M., Chan, P.A., 2020. Adapting Substance Use Treatment for HIV Affected Communities During COVID-19: Comparisons Between a Sexually Transmitted Infections (STI) Clinic and a Local Community Based Organization. AIDS Behav 24, 2999–3002. https://doi.org/10.1007/s10461-020-02933-y

SAMHSA, 2023. Waiver Elimination (MAT Act) [WWW Document]. URL https://www.samhsa.gov/medications-substance-use-disorders/waiver-elimination-mat-act (accessed 7.24.23).

SAMHSA, 2018. Key Substance Use and Mental Health Indicators in the United States: Results from the 2018 National Survey on Drug Use and Health. Substance Abuse and Mental Health Services Administration, Rockville, MD.

Sanders, J.N., Glynn, T.R., Mayo, D., Dale, S.K., Harkness, A., Safren, S.A., 2023. Does Problematic Substance Use Moderate Efficacy of Cognitive Behavioral Therapy for Adherence and Depression in HIV? AIDS Behav 27, 1123–1132. https://doi.org/10.1007/s10461-022-03849-5

Schranz, A.J., Davy-Mendez, T., Eron, J.J., Napravnik, S., 2021. Opioid misuse among persons with HIV engaged in care in the Southeastern US. AIDS Care 33, 148–153. https://doi.org/10.1080/09540121.2019.1699644

Smith, J.D., Li, D.H., Rafferty, M.R., 2020. The Implementation Research Logic Model: a method for planning, executing, reporting, and synthesizing implementation projects. Implementation Science 15, 84. https://doi.org/10.1186/s13012-020-01041-8

Thomas, C.P., Fullerton, C.A., Kim, M., Montejano, L., Lyman, D.R., Dougherty, R.H., Daniels, A.S., Ghose, S.S., Delphin-Rittmon, M.E., 2014. Medication-Assisted Treatment With Buprenorphine: Assessing the Evidence. PS 65, 158–170. https://doi.org/10.1176/appi.ps.201300256

U.S. Department of Health & Human Services, 2022. Biden Administration Announces $1.5 Billion Funding Opportunity for State Opioid Response Grant Program [WWW

Document]. HHS.gov. URL https://www.hhs.gov/about/news/2022/05/19/biden-administration-announces-15-billion-funding-opportunity-state-opioid-response-grant-program.html (accessed 7.24.23).

Waters, R.C., Oviedo-Joekes, E., Rawlins, J., 2021. HIV and overdoses: diversifying therapies for opioid use disorder. The Lancet 398, 741–742. https://doi.org/10.1016/S0140-6736(21)01251-4

Wen, H., Xiang, X., Jiang, Y., Zhang, H., Zhang, P., Chen, R., Wei, X., Dong, Y., Xiao, S., Lu, L., 2023. Comparative efficacy of psychosocial interventions for opioid-dependent people receiving methadone maintenance treatment: A network meta-analysis. Addiction. https://doi.org/10.1111/add.16167

Wyse, J.J., McGinnis, K.A., Edelman, E.J., Gordon, A.J., Manhapra, A., Fiellin, D.A., Moore, B.A., Korthuis, P.T., Kennedy, A.J., Oldfield, B.J., Gaither, J.R., Gordon, K.S., Skanderson, M., Barry, D.T., Bryant, K., Crystal, S., Justice, A.C., Kraemer, K.L., 2022. Twelve-Month Retention in Opioid Agonist Treatment for Opioid Use Disorder Among Patients With and Without HIV. AIDS Behav 26, 975–985. https://doi.org/10.1007/s10461-021-03452-0

Yamamoto, A., Needleman, J., Gelberg, L., Kominski, G., Shoptaw, S., Tsugawa, Y., 2019. Association between Homelessness and Opioid Overdose and Opioid-related Hospital Admissions/Emergency Department Visits. Soc Sci Med 242, 112585. https://doi.org/10.1016/j.socscimed.2019.112585
