# Supplementary material for: Provider perspectives on screening and treatment for opioid use disorder and mental health in HIV care: A qualitative study
Source: PLoS One. 2024 Jun 24;19(6):e0305174. doi: 10.1371/journal.pone.0305174 (PMC11195992; doi:10.1371/journal.pone.0305174)
Supplement: Attachment — Submitted filename: Response to Reviewers.docx [file pone.0305174.s002.docx]

**Response to Reviewers**

**Reviewer #1:** This is an interesting study. The paper is well written and structured. However, the results do not clearly answer the aims of the study as well as are not quite related to the topic. The results showed that (1) the current lack of OUD and mental health screening and treatment in HIV care facilities, (2) situation of substance use among HIV patient is high, but opioid use was emphasized less often, (3) barriers to improve HIV patient care were, patient with mental health issue(s), financial and social challenges, no stable housing, transportation, stigma of mental health and substance use, limitation of referral system; which look like general barrier for HIV care (with or without any comorbidity). As mentioned before, the results do not clearly show barriers and strategies for the implementation of screening and treatment of OUD and metal health issues. Moreover, in the discussion part, some details seem over-mentioned from those reported in the results section. For example, strong consensus, rapid increase of OUD.

Thank you for these helpful comments. We agree with the reviewer that title and aims of the study did not reflect the full range of findings. We have now revised the title and the objectives of the study (last paragraph of the Introduction) to encompass the full range of study findings: “The objectives of this study were to further explore the current landscape of screening and treatment for OUD and mental health challenges in HIV care, including potential benefits and barriers related to the implementation of opioid integrated screening and treatment.”

We also softened some of the language in the Discussion as requested by the reviewer to better reflect the views of all participants, for example changing “strong consensus” to “general agreement” and “rapidly increasing” to “increasing”.

Abstract
- The authors said that in-depth interviews were conducted with 21 HIV treatment providers and social services providers; however, in the METHODS section showed individual semi-structure interview and group interview. The authors should provide more specific information about the method used in the abstract.

We have added “12 individual interviews and 1 group interview with 9 participants” to the abstract to clarify this point.

- May add sample recruitment method and study setting in the abstract.

We added the sentence, “Providers were purposively recruited from known clinics in Mecklenburg County, North Carolina, U.S.A.”

- The results did not clearly answer the aims to the study.

See response to this point above.

Introduction
- May add more information/references regarding strategies and barriers of integrating screening and treatment of OUD and mental health in HIV patients.

Thank you for this recommendation. We have expanded Introduction paragraph 5 (bottom of page 5) with additional information and references related to this point.

Methods:
- The authors reported that purposive samples were recruited, please provide more specific characteristics of sample that will be used to recruit samples.

We have added more detail related to our recruitment strategy in the first paragraph of the methods section on page 6.

- The interview guides did not really show major questions that were used on the interview, only prompts are shown. Moreover, there were around 30 prompt questions in the table, is 30-60 minutes enough to obtain details and rich understanding of the topic?

We now clarify on page 7 that Table 1 is a summary of the interview guide and not the entire interview guide. Many prompts/questions did not apply to all participants and were skipped when not applicable, which explains the variability in interview length and the reason we were able to complete the interviews within the allotted time with excellent detail/richness. We now clarify on Page 7, “In instances where an area of inquiry did not apply to an interviewee, that section was skipped.”

Results:
- Organizational characteristics: the theme seem to present characteristics of HIV care facilities, however, the data only showed provided services of the facilities. The authors may present more characteristics of the facilities (such as number of health care providers/specialists, number of staffs) and relation of organizational characteristics and provided services (if possible)

We agree that number of total providers and size of the facilities could have provided an interesting point of comparison. However, given the small number of facilities in this county, we were concerned that adding too many details about the facilities could jeopardize the anonymity of the facility and its providers. We have added the following sentence in the limitations paragraph to acknowledge the importance of this information in future studies: “Future analyses may wish to assess differences in service provision based on size and type of facility.”

Discussion
- 3rd Paragraph, the authors said that there was a rapidly increasing burden of OUD in HIV patients, but in the result part stated that “opioids were emphasized less often”

We have softened the language in this sentence as noted above. Additionally, this sentence is referring to the increased burden of OUD in clinics where screening and services are provided, as noted on pages 11-12: “A provider at this clinic noted ‘increasing amounts of substance use in general and increasing demand for our MAT program,’ with 30% growth in their MAT patient population in the prior year. As demand has increased, they have hired more providers capable of providing MAT to help meet this need.” We clarified this point with a new sentence and citation in the discussion section on page 18: “Providers who did not conduct routine screening rarely asked about opioid use in their informal substance use assessments, reinforcing OUD’s status as a hidden epidemic in many healthcare settings (Limratana and Yuki, 2023).”

- Please discuss the limitation of the study regarding conducted both individual and group interviews.

We added the following sentence in the limitations paragraph: “Researchers may wish to examine potential differences in themes observed in individual versus group interview formats.”

Reviewer #2: The manuscript is well written and the few corrections identified are given as comments in the attached manuscript.

Table 2: “Total (%) is 101%. Maybe N=6 can be reduced to 28% to round off to 100% for race as well.”

We have chosen not to artificially round down these percentages from 29% to 28% (6/21 = 28.57%) as we believe most readers will understand the cumulative percentage of 101% is due to a rounding issue.

The reference section needs to be verified with reference to the journal referencing guidelines for maximum authors and reference style.

We have now revised the reference style to the PLOS One format, with citation numbers in brackets and maximum of 6 authors listed per reference.

Reviewer #3: The authors aimed to explore the potential benefits, barriers, and strategies for implementing opioid screening and treatment, along with mental health treatment, in HIV care. The methodology employed is transparent and valid for the stated objective. The findings of the manuscript are of interest to the readers of the Journal. However, I have the following minor concerns regarding the study results and title:

Comment:

1. The title of the study is "Exploring strategies for integrating screening and treatment for opioid use disorder and mental health in HIV Care: A qualitative study with providers." However, the study results do not primarily focus on strategies; instead, they emphasize identifying barriers. Therefore, it is recommended to revise the title to "Identification of barriers for integrating screening and treatment for opioid use disorder and mental health in HIV Care: A qualitative study with providers." Similarly, the objectives should be adjusted to remove references to strategies. Alternatively, the theme of strategies could be addressed in the results section and discussed in detail.
We have revised the title accordingly and removed references to strategies, as

described in our response to reviewer 1.

2. In Table 2, the age unit (Age in years) should be mentioned.

Thank you. This has been added.
