# Supplementary material for: Provider perspectives on screening and treatment for opioid use disorder and mental health in HIV care: A qualitative study
Source: PLoS One. 2024 Jun 24;19(6):e0305174. doi: 10.1371/journal.pone.0305174 (PMC11195992; doi:10.1371/journal.pone.0305174)
Supplement: Attachment — Submitted filename: Response to Reviewers_R2.docx [file pone.0305174.s004.docx]

**Response to Reviewers**

Who were the other professionals? Medical referred to medical or social service provider or vice versa? or Medical to nurse?

We note clarify on Page 6 that participants referred both “medical and social services providers” to participate in the study.

How long this group interview was conducted?

We now clarify on Page 7 that the group interview was 35 minutes long.
